# High-Quality Draft Genome Sequence of *Bacillus amyloliquefaciens* Strain 629, an Endophyte from *Theobroma cacao*

**DOI:** 10.1128/genomeA.01325-15

**Published:** 2015-11-19

**Authors:** Brena M. M. SantAnna, Phellippe P. A. Marbach, Marcelo Rojas-Herrera, Jorge T. De Souza, Milton R. A. Roque, Artur T. L. Queiroz

**Affiliations:** a Universidade Federal da Bahia (UFBA), Salvador, Brazil; b Universidade Federal do Recôncavo da Bahia (UFRB), Cruz das Almas, Brazil; c Centro de Genómica y Bioinformática, Universidade Mayor, Santiago, Chile; d Universidade Federal de Lavras (UFLA), Lavras, Brazil; e Centro de Pesquisas Gonçalo Moniz (CPqGM)-FIOCRUZ, Salvador, Brazil

## Abstract

*Bacillus amyloliquefaciens* strain 629 is an endophyte isolated from *Theobroma cacao* L. Here, we report the draft genome sequence (3.9 Mb) of *B. amyloliquefaciens* strain 629 containing 16 contigs (3,903,367 bp), 3,912 coding sequences, and an average 46.5% G+C content.

## GENOME ANNOUNCEMENT

Bacilli are frequently isolated as endophytes and are common components of the microbiota of several plant species (1, 2). Strain 629 was isolated from a healthy *Theobroma cacao* tree and was initially identified as *Bacillus subtilis* (3), but further analysis based on *gyr*B and *rec*A sequences revealed that its true identity is *Bacillus amyloliquefaciens* (4). This isolate colonizes different host and plant tissues under both sterile and nonsterile conditions and promotes plant growth (3, 4). Strain 629 produces the lipopeptides iturin, fengicin, and surfactin and volatile organic compounds that may be active in the biocontrol of several fungal plant pathogens (unpublished data) and pathogenic bacteria, including *Curtobacterium flaccumfaciens* pv. flaccumfaciens (5). Furthermore, *B. amyloliquefaciens* 629 is currently being used as a model to study endophytic colonization (4). This strain is deposited in the Biological Institute Culture Collection of Phytopathogenic Bacteria (IBSBF) (Campinas, São Paulo, Brazil) under accession no. IBSBF-3106. This collection is registered with the World Data Centre for Microorganisms collection under no. WDCM-110.

Genomic DNA from isolate 629 was extracted and sequenced using the Ion Torrent PGM platform (Life Technologies) 318 chip. A total of 7,567,586 reads with an average length of 330 pb were obtained. All reads were assembled to an initial draft genome of 3,866,991 nucleotides at 443-fold coverage using the SPAdes Genome Assembler version 3.5.0, generating 129 unoriented contigs, with a G+C content of 46.5%, (*N*_50_: 285,363 bp).

Contigs were ordered using CONTIGuator 2.3 (http://contiguator.sourceforge.net*/*) (6) with the *B. Amyloliquefaciens* CC178 genome, the closest available, as a reference (GenBank accession no. CP006845.1). Subsequently, 34 contigs with 3.8 Mb were aligned with the reference genome to order the contigs. A total of 95 contigs (only 9 > 600 bp) corresponding to 29,876 nucleotides were not mapped to the reference genome. These sequences were identified as redundant contigs, according to BLAST results, and were removed from the assembly. To solve the repetitive sequences and the remaining gaps the MapRepeat pipeline (7) was used, resulting in the final high-quality draft genome sequence with 16 contigs, containing 3,903,367 bp.

Genome annotation was performed with RAST version 2.0 server (8). The genome of strain 629 is composed of 4,013 predicted genes, including 3,912 protein-coding sequences, 82 tRNAs, and 19 copies of the genes for 5S, 16S, and 23S rRNA. The genome of strain 629 is closely related to that of *B. amyloliquefaciens* CC178 with an identity of 99% (97% coverage) and also has a similar numbers of predicted genes (9).

Subsequent analysis of the genome content of *B. amyloliquefaciens* 629 and its comparison with phylogenetically related strains will help to determine key aspects of its interaction with the environment, plants, and other microorganisms.

### Nucleotide sequence accession numbers.

The *Bacillus amyloliquefaciens* strain 629 whole-genome shotgun (WGS) project has been deposited at DDBJ/EMBL/GenBank under the accession no. LGYP00000000. The version described in this paper is the first version, LGYP01000000, and consists of sequences LGYP01000001 to LGYP01000016.
